# In this issue of Current Oncology

**Published:** 2009-08

**Authors:** M. McLean

In Canada, many patients undergoing cancer treatment are faced with exceptional costs—because of travel and related expenses, for instance—not always experienced in other jurisdictions. In a review titled “Perceptions of health care providers concerning patient and health care provider strategies to limit out-of-pocket costs for cancer care,” Dr. Maria Matthews and colleagues look at these “soft” costs from the perspective of patients in Canada’s most easterly province. The article sets out some of the strategies that cancer patients and their caregivers are both forced to use to limit the burden, which moreover falls somewhat disproportionately on rural as compared with urban patients.

The guest editorial in this issue of *Current Oncology* finds Dr. Louis Touyz questioning the rationale of immunizing only girls against the human papilloma virus; and in a not-dissimilar vein, Drs. Joshua Uronis and Christian Jobin take note of the 100 trillion bacterial and like particles in the “average” colon and review the evidence for colon-based microorganism-mediated signals for tumour suppression and promotion.

In the rapidly changing field of advanced non-small-cell lung cancer, “responsive clinicians face difficult tradeoffs as they try to balance the pros and cons of early adoption [of new therapies] with excessive conservatism.” Dr. Mark Vincent invites us to peruse a “personal view of how to navigate these waters,” which “although ... written especially for patients who like to be the captain of their own ship, [holds] good reason to believe that all patients will eventually be managed by similar, if not identical, means.” In the equally rapidly changing field of metastatic breast cancer, our Practice Guideline series reviews the role of her2-targeted therapies in women with her2-overexpressing metastatic breast cancer.

Elsewhere in the issue, the role of rna in promoting and regulating cancer is comprehensively reviewed by Drs. Thomas Duchaine and Frank Slack. This topic area is potentially of huge importance not only for understanding this microenvironment, but also for the development of additional targeted cancer therapies.

Strong representation from Canadian radiation centres in *Current Oncology* continues with an overview of the results of bladder-conserving cancer therapies. Dr. Luis Souhami and colleagues closely scrutinize a strategy, largely developed and promoted within Canada, that takes a trimodal approach to the management of muscle-invasive disease. Although most bladder cancers are superficial at presentation, disease at this site is the ninth most frequent cancer seen worldwide.

The journal continues to expand its hybrid concept for some manuscripts of a more specialized nature. Expanded abstracts of these papers appear in the print issue with links to the full versions published online. This presentation achieves a secondary benefit of more speedy publication. Printing *Current Oncology* is a costly venture, and we believe that the hybrid format is in the long-term best interest not only of the journal, but also of our readers and contributors.

We trust that readers will find all of this issue’s articles enlightening and thought-provoking.

